# HLA incompatibility mitigation strategies in off-the-shelf cancer immunotherapies: clinical implications and a practical framework for strategy selection and combination

**DOI:** 10.3389/fimmu.2026.1803331

**Published:** 2026-04-17

**Authors:** Rajdeep Das, Medhat Askar

**Affiliations:** 1 Department of Pathology, University Hospitals Cleveland Medical Center, Cleveland, OH, United States; 2 Case Western Reserve University School of Medicine, Cleveland, OH, United States; 3 Division of Hematology and Cellular Therapy, Wesley Center for Immunotherapy UH Seidman Cancer Center, Cleveland, OH, United States; 4 Department of Pathology and Laboratory Medicine, Tufts Medicine and Tufts Medical Center, Boston, MA, United States; 5 Department of Pathology and Laboratory Medicine, Baylor University Medical Center, Dallas, TX, United States; 6 Clinical Services, NMDP, Minneapolis, MN, United States

**Keywords:** allogeneic cell therapy, cancer immunotherapy, HLA incompatibility, immunogenetics, population-informed matching

## Introduction

Allogeneic “off-the-shelf” cellular immunotherapies are rapidly transforming the landscape of cancer treatment. Platforms including allogeneic CAR-T cells, CAR-NK cells, TCR-engineered lymphocytes, and induced pluripotent stem cell (iPSC)–derived immune effectors are designed to overcome the manufacturing delays, high costs, and scalability constraints inherent to autologous therapies (1–5). Their immediate availability is particularly advantageous for patients with aggressive or relapsed malignancies, as demonstrated by early clinical experience with allogeneic CAR-NK and iPSC-derived immune cell platforms (6–8).

Cancer immune surveillance is a vital physiological process wherein the immune system continuously monitors, recognizes, and eliminates nascent malignant cells before they develop into clinically apparent tumors (9). This dynamic interaction relies fundamentally on the antigen presentation machinery, particularly the highly diverse Human Leukocyte Antigen (HLA) system, encoded by genes in the Major Histocompatibility Complex (MHC). HLA class I and class II molecules act as critical cellular display windows, presenting tumor-associated antigens and neoantigens to activate cytotoxic CD8+ T cells and helper CD4+ T cells, respectively (10). The immense genetic diversity of HLA alleles ensures that a broad spectrum of unique tumor antigens can be recognized, igniting robust anti-tumor responses (11). The efficacy of this surveillance network is intricately linked to cellular stress responses—such as DNA damage, oxidative stress, and cellular senescence—which can act as danger signals that alert immune sentinels to the presence of abnormal cells (12, 13).

Despite these advantages, immune-mediated rejection remains the dominant biological barrier limiting durability, efficacy, and scalability of allogeneic cancer immunotherapies (1, 14). Central to this challenge is incompatibility within the human leukocyte antigen (HLA) system, which governs host–donor immune recognition (15). While tumor antigen selection and receptor engineering have advanced rapidly, host–donor immunogenetic incompatibility has proven more difficult to overcome and remains a key determinant of therapeutic persistence, patient eligibility, and trial feasibility (1, 15, 16).

The importance of the HLA system in cancer immunotherapy stems from its central role in antigen presentation. HLA class I molecules present peptide fragments derived from intracellular proteins on the cell surface, enabling CD8^+^ T cells to recognize infected or transformed cells (17). This principle of major histocompatibility complex (MHC) restriction, first established by Zinkernagel and Doherty, provided the conceptual basis for antigen-specific T-cell recognition in cancer immunology (18). Landmark studies subsequently demonstrated that human tumors express antigenic peptides recognizable by T cells in an HLA-restricted manner, thereby laying the foundation for modern cancer immunotherapy (19, 20). Tumor-associated antigens are typically non-mutated self-antigens that are aberrantly expressed or overexpressed in malignant cells, whereas tumor-specific antigens arise from somatic alterations and may offer greater therapeutic specificity. Because each HLA allele binds peptides with distinct sequence motifs, variability in HLA alleles across individuals and populations influences which antigens can be effectively targeted by T-cell–based therapies (11).

Understanding these cardinal principles of immune surveillance and the diverse mechanisms of immune escape is crucial. It not only elucidates the epidemiological links between chronic inflammation, aging, and cancer risk but also provides the mechanistic foundation for developing next-generation immunotherapies aimed at restoring and unleashing the body’s innate anti-tumor immunity (21, 22). Importantly, HLA incompatibility directly constrains patient access. Allele-restricted approaches—most commonly targeting HLA-A*02:01—exclude many patients before treatment response can be assessed and may disproportionately affect genetically diverse and underrepresented populations because HLA allele frequencies vary substantially across ancestral and geographic groups (16, 23, 24). As off-the-shelf therapies advance into early- and mid-phase trials, insufficient mitigation of HLA incompatibility risks limiting real-world adoption and exacerbating disparities in access to advanced cancer therapeutics (16, 24).

Rather than treating incompatibility as a downstream complication, we argue that HLA coverage should be considered an explicit and controllable design variable. Here, we propose a practical framework to guide the rational selection and combination of strategies aimed at expanding HLA coverage in off-the-shelf cancer immunotherapies.

## HLA coverage as a translational bottleneck

Many contemporary allogeneic cancer immunotherapy platforms rely on narrow HLA restrictions to simplify early development, most often focusing on HLA-A*02:01 (25). While expedient, this approach limits the proportion of eligible patients and introduces predictable racial and ethnic disparities due to population-level variation in HLA allele frequencies (16, 24). Because eligibility is determined upstream of therapeutic exposure, excluded patients are unable to benefit regardless of biological potency or clinical promise (16).

Beyond eligibility constraints, HLA mismatch contributes directly to host-mediated immune rejection that undermines persistence and scalability—the central value proposition of off-the-shelf therapies (26). These challenges parallel long-recognized limitations in allogeneic hematopoietic stem cell transplantation, where donor availability, graft survival, and outcomes are strongly influenced by HLA diversity and race-associated matching constraints (27, 28). As allogeneic immunotherapies progress toward later-phase trials and broader clinical deployment, HLA coverage has shifted from a theoretical concern to a defining translational bottleneck that shapes feasibility, durability, and equity (25, 26).

## A practical framework for expanding HLA coverage

**Figure 1:**
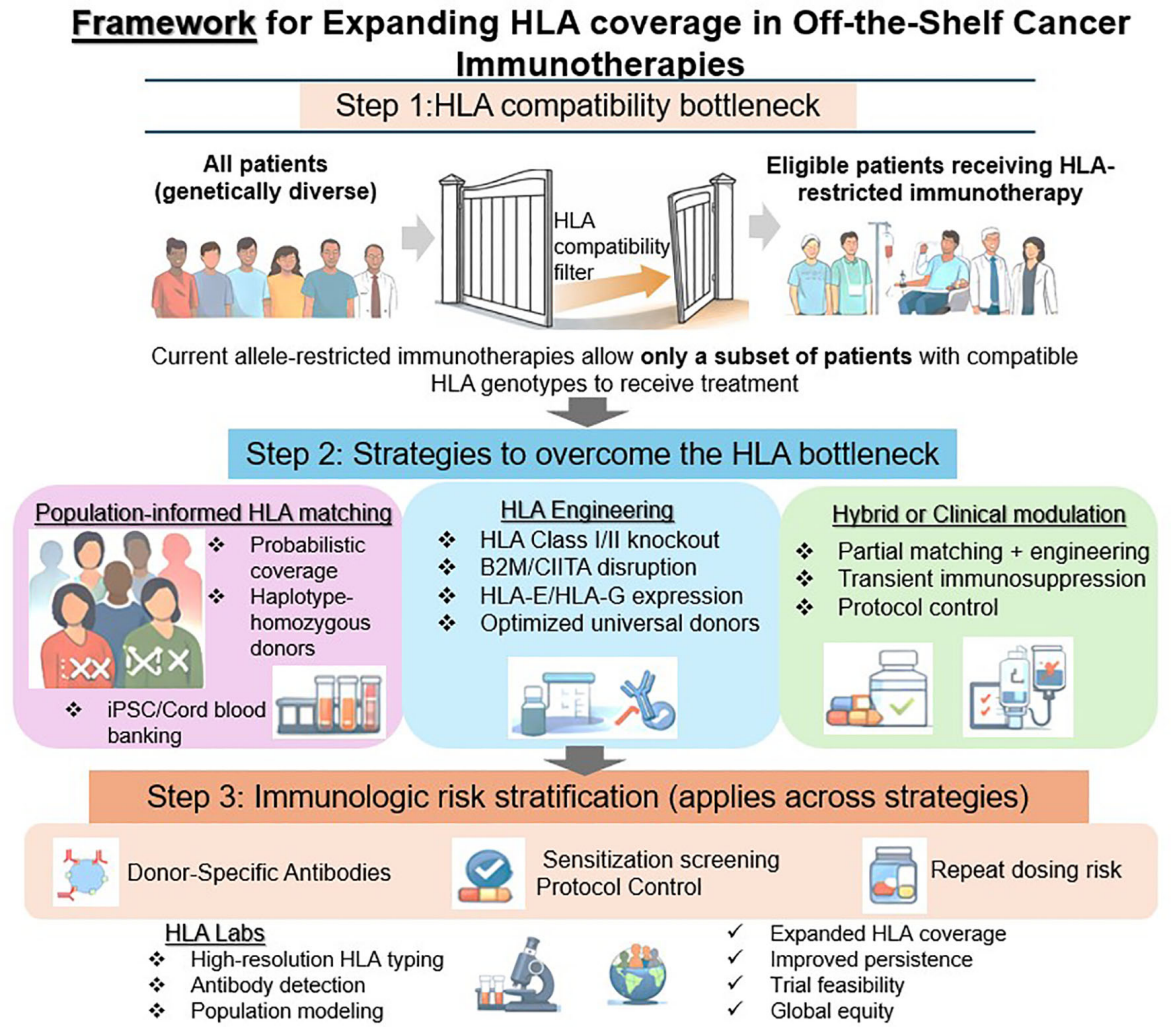
Framework for expanding HLA coverage in off-the-shelf cancer immunotherapies: In the schematic, the diverse patient icons on the left represent the genetically heterogeneous patient population, the narrowing gateway represents the HLA compatibility barrier imposed by allele-restricted immunotherapies, and the expanded pathway on the right represents strategies intended to broaden patient eligibility. Step 1 illustrates the HLA compatibility bottleneck, where genetically diverse patient populations are narrowed to a smaller subset eligible for HLA-restricted immunotherapies. Step 2 summarizes three complementary strategy domains designed to overcome this bottleneck: (1) population-informed HLA matching, which leverages population allele frequencies and haplotype-homozygous donors; (2) HLA engineering approaches aimed at reducing immunogenicity through modification of HLA class I/II pathways; and (3) hybrid or clinical modulation strategies including partial matching, conditioning regimens, and protocol-level adaptations. Step 3 highlights immunologic risk stratification, including donor-specific antibody screening and sensitization assessment, which applies across strategies to guide patient selection and treatment feasibility.

No single strategy fully resolves HLA-related barriers across all disease contexts and therapeutic platforms. Instead, we organize mitigation approaches into four complementary strategy domains that address distinct but overlapping aspects of HLA incompatibility: (1) population-informed matching approaches, (2) immunogenicity reduction through HLA engineering, (3) hybrid and clinical modulation strategies, and (4) immunologic risk stratification. These domains can be applied independently or in combination to tailor allogeneic therapies to specific persistence requirements, safety considerations, and target populations. This framework is summarized schematically in **Figure 1**, which illustrates how population-informed matching, HLA engineering, and hybrid clinical modulation strategies are integrated with immunologic risk stratification to expand HLA coverage in off-the-shelf cancer immunotherapies.

## Strategy domain 1: population-informed matching approaches

Population-informed matching strategies aim to maximize partial HLA compatibility at scale by leveraging population-level HLA allele and haplotype frequency data to prioritize donors or cell lines capable of covering large proportions of a target population. In this context, population-informed matching refers to the use of HLA frequency distributions across populations to guide donor selection, cell-bank composition, and target prioritization in ways that maximize projected patient coverage. One prominent example is the use of haplotype-homozygous donors to generate cellular products, particularly iPSC-derived immune effectors, enabling a limited number of cell lines to provide partial matching for a substantial proportion of a given population (29, 30). Modeling studies demonstrate that carefully selected haplotypes can extend coverage while preserving manufacturing scalability (31).

Cord blood–derived cells are particularly attractive in this context. Cord blood units are widely banked, rigorously HLA typed, and exhibit greater tolerance of HLA mismatch compared with adult peripheral blood–derived cells, reflecting differences in immune maturity and alloreactivity (32). These characteristics have supported long-standing clinical use in mismatched transplantation and motivate ongoing efforts to define haplotype panels that optimize regional or population-specific coverage (30).

In allogeneic cancer immunotherapy, population-informed matching is best suited for settings in which partial compatibility improves persistence without requiring full matching, such as repeat dosing strategies or therapies requiring prolonged *in vivo* activity (3). However, even optimized haplotype panels provide incomplete coverage in genetically diverse populations and do not eliminate alloimmune responses driven by additional loci or minor histocompatibility antigens (29, 31). As such, these approaches reduce—but do not abolish—immunologic risk.

## Strategy domain 2: immunogenicity reduction through HLA engineering

An alternative approach involves direct genetic modification to reduce immune recognition. Advances in genome editing and RNA-based technologies enable disruption of classical HLA class I and class II pathways—commonly via B2M or CIITA—often combined with expression of non-polymorphic inhibitory molecules such as HLA-E or HLA-G to mitigate NK cell–mediated “missing self” responses (33–36). In addition to permanent gene disruption, short hairpin RNA (shRNA)–based approaches have been employed to selectively downregulate classical HLA class I expression while preserving inhibitory signaling, offering a potentially reversible and tunable method of immunogenicity reduction (37).

Hypoimmunogenic engineering offers the theoretical advantage of creating near-universal donor products unconstrained by allele frequency, thereby decoupling eligibility from population HLA distribution. This strategy has gained momentum in iPSC-derived platforms and broader immune-evasive cell therapy concepts, with preclinical and translational studies demonstrating durable immune evasion through combinatorial targeting of adaptive and innate immune pathways (34, 35, 38).

However, extensive HLA abrogation introduces new considerations. Loss of classical HLA expression can increase susceptibility to NK-cell attack, infection, or impaired immune surveillance, necessitating additional engineering safeguards (33, 39). Accordingly, HLA engineering should be framed not simply as HLA deletion, but as controlled immunologic redesign intended to balance evasion of host rejection with preservation of safety-relevant immune functions. More broadly, immune-evasive designs raise long-term safety questions, including potential oncogenic or infectious risks, that require indication-specific risk–benefit assessment rather than uniform application (35, 39).

## Strategy domain 3: hybrid and clinical modulation strategies

While population matching and HLA engineering address immunogenicity at the platform level, hybrid strategies operate at the patient or protocol level (An et al., 2023). Recognizing the limitations of single-strategy solutions, integrated approaches combining partial HLA compatibility with targeted engineering or clinical immunomodulation are increasingly explored (1, 6, 40).

Short-term immunosuppressive or lymphodepleting conditioning regimens can transiently attenuate host T- and NK-cell–mediated rejection, improving early persistence of HLA-mismatched cellular products (14). Such strategies may be particularly relevant when partial compatibility reduces—but does not eliminate—immune recognition and when therapeutic efficacy depends on sustained cellular persistence. Importantly, the intensity, timing, and duration of conditioning can be modulated to balance persistence benefits against toxicity and infection risk.

Hybrid strategies emphasize flexibility, positioning clinical trial design itself as an active lever for managing HLA-related risk. Variables such as conditioning intensity, infusion scheduling, repeat dosing, and adjunctive immunomodulation can be adjusted to align immunogenicity mitigation with therapeutic goals. In this context, HLA incompatibility is no longer a fixed constraint but a parameter that can be partially managed through protocol design (1, 5). This domain is particularly relevant in early-phase development, where protocol-level adaptation may provide a pragmatic bridge between proof-of-concept efficacy and more durable platform-level solutions.

## Strategy domain 4: immunologic risk stratification and donor-specific antibodies

Distinct from strategies targeting cell-mediated rejection, immunologic risk stratification addresses humoral alloimmunity. Donor-specific antibodies (DSA) are well-established predictors of graft failure and adverse outcomes in solid organ transplantation and hematopoietic cell transplantation (41, 42), and expert consensus increasingly emphasizes their relevance in mismatched settings (43).

In contrast, the role of DSA in allogeneic cancer immunotherapy remains less well defined, despite growing recognition that humoral sensitization may limit persistence and efficacy (44). Importantly, DSA screening is inherently strategy-agnostic and can be applied regardless of upstream HLA mitigation approach.

Within this framework, immunologic risk stratification functions as a cross-cutting layer of evaluation, particularly relevant for therapies requiring durable persistence or repeat dosing. Transplantation experience provides practical guidance on antibody specificity, strength thresholds, and mitigation strategies—such as patient selection or desensitization—that may be adapted as allogeneic cancer immunotherapy platforms mature (41, 43, 45). In this sense, immunologic risk stratification does not replace upstream mitigation strategies, but rather refines their clinical application by identifying settings in which residual alloimmune risk is likely to be acceptable, manageable, or prohibitive.

## Operationalizing the framework: integrating immunogenetics into allogeneic cell therapy development

Operationalizing strategies to expand HLA coverage requires systematic integration of immunogenetic expertise across the development lifecycle of allogeneic cell therapies. Histocompatibility and immunogenetics laboratories are uniquely positioned to support this integration through high-resolution HLA typing, population-level allele and haplotype frequency analysis, donor-specific antibody screening, and functional assessment of alloimmune risk. These capabilities directly inform platform design, donor or cell line selection, and eligibility criteria, enabling prospective, data-driven decisions rather than *post hoc* exclusion (11, 46).

In addition, early-stage target selection increasingly depends on accurate characterization of the HLA ligandome and of tumor-associated versus tumor-specific antigens that can be reproducibly presented across relevant patient subsets (11, 20, 47). Likewise, choice of effector platforms including allogeneic T cells, NK cells, and iPSC-derived immune effectors—intersects directly with HLA biology because each platform differs in its dependence on antigen presentation, persistence requirements, and susceptibility to host rejection (1, 3, 4, 19).

Incorporating immunogenetic data early—during target selection, product engineering, and clinical trial design—can improve enrollment efficiency, reduce protocol amendments, and enhance interpretability of clinical outcomes (1, 2, 48). Moving HLA considerations upstream also promotes transparency, allowing trial sponsors, regulators, and patients to better understand the scope, assumptions, and limitations of product coverage.

As off-the-shelf platforms advance toward later-phase trials and broader clinical deployment, standardized reporting of HLA assumptions, allele coverage, and immunologic risk mitigation strategies will become increasingly important. Such transparency will facilitate regulatory evaluation, support cross-trial comparisons, and ensure that scalability and equity are assessed alongside efficacy and safety. Embedding immunogenetics as a core component of allogeneic cell therapy development thus represents not merely technical refinement, but foundational infrastructure for responsible translation (49, 50).

## Discussion and outlook

Off-the-shelf cancer immunotherapies hold substantial promise to transform cancer care by enabling rapid, scalable, and broadly accessible treatment. However, this promise remains fundamentally constrained by HLA incompatibility, which limits patient eligibility, compromises persistence, and risks perpetuating longstanding disparities in access to advanced therapies.

The framework outlined here positions HLA coverage as a controllable design variable rather than an unavoidable limitation. By integrating population-informed matching, immunogenicity reduction through HLA engineering, clinical modulation strategies, and immunologic risk stratification, developers can rationally tailor allogeneic platforms to specific disease contexts, persistence requirements, and target populations. No single strategy is universally optimal; thoughtful combination and context-dependent selection are essential.

Looking forward, early and systematic integration of immunogenetic expertise will be critical to realizing the full potential of allogeneic cell therapies. As the field matures, success will increasingly be measured not only by biological efficacy, but also by scalability, reproducibility, and equitable impact across diverse populations. Treating HLA compatibility as prerequisite infrastructure—rather than downstream optimization—provides a practical pathway toward inclusive, durable, and globally relevant next-generation cancer immunotherapies.
